# A Meta-Analysis of the Effects of Tai Chi on Glucose and Lipid Metabolism in Middle-Aged and Elderly Diabetic Patients: Evidence from Randomized Controlled Trials

**DOI:** 10.1155/2021/6699935

**Published:** 2021-03-22

**Authors:** Ya-nv Liu, Lin Wang, Xin Fan, Shijie Liu, Qi Wu, You-Ling Qian

**Affiliations:** ^1^Department of Physical Education, Wuhan University of Technology, Wuhan 430070, China; ^2^School of Physical Education, Hubei Normal University, Huangshi 435002, China; ^3^School of Physical Education, Jianghan University, Wuhan 430056, China; ^4^School of Physical Education, Hubei Minzu University, Enshi 445000, China; ^5^Graduate School, Adamson University, Manila 1000, Philippines

## Abstract

This research review aimed to evaluate the effect of practicing Tai Chi on glucose and lipid metabolism in middle-aged and elderly diabetic patients. Furthermore, it aimed to provide a theoretical basis for the practice of Tai Chi as a way to improve glucose and lipid metabolism in middle-aged and elderly diabetic patients. Therefore, we searched for randomized controlled trials on the practice of Tai Chi in middle-aged and elderly diabetic patients in Chinese- and English-language electronic databases, such as Web of Science, PubMed, the Cochrane Library, EMBASE, Google Scholar, CNKI, Wanfang Database, and Weipu. We collected articles published no later than August 1, 2020. The methodological quality of the included studies was evaluated according to the standards of the Cochrane Collaboration System Evaluation Manual (version 5.1.0). Finally, 14 articles were included, showing an average Physiotherapy Evidence Database scale score of 6.57. The articles were meta-analyzed using Stata 14.0 software, showing that practicing Tai Chi improved middle-aged and elderly diabetic patients' fasting blood glucose (WMD = −0.60, 95% CI [−1.08, −0.12], *p*=0.015), glycosylated hemoglobin (WMD = −0.87, 95% CI [−1.60, −0.14], *p*=0.019), total cholesterol (WMD = −0.48, 95% CI [−0.83, −0.14], *p*=0.006), triglycerides (WMD = −0.21, 95% CI [−0.37, −0.04], *p*=0.014), and low-density lipoprotein cholesterol level significantly (WMD = −0.32, 95% CI [−0.63,−0.00], *p*=0.050). Conversely, patients' high-density lipoprotein cholesterol levels (WMD = 0.09, 95% CI [−0.01, 0.17], *p*=0.136) showed no obvious improvement. In conclusion, practicing Tai Chi in sessions lasting longer than 50 minutes (at least three times per week, for at least 12 weeks) can effectively improve glucose and lipid metabolism in middle-aged and elderly diabetic patients. However, several other factors affect glucose and lipid metabolism; therefore, further high-quality research is needed. Protocol registration number: INPLASY2020120107.

## 1. Introduction

In 2007, the American Academy of Sports Medicine and the American Medical Association jointly launched the *Exercise is Medicine* initiative in the United States. Taken as an academic philosophy and health-promotion initiative, the concept of exercise as medicine was received enthusiastically in many countries. The main goals of this initiative were the following: To provide professional training programs and continuing education programs for primary-care doctors and nurses (to ensure that they fully understand the importance of sports for health self-management), and to improve the overall national health level and happiness index through exercise prescriptions formulated by professional health-care personnel [1].

The concept of exercise as medicine is based on the findings of several previous studies showing that exercise ameliorates chronic disease. For example, Igarashi et al. found that water sports improved blood pressure in patients with hypertension [2]. Furthermore, Nalbant et al. implemented a six-month aerobic intervention for elderly patients with chronic obstructive pulmonary disease and found that their lung function and lower limb strength were significantly improved [3]. Additionally, Wang et al. found that practicing yoga promoted the recovery of stroke patients' neurological functions and improved their overall health [4].

Diabetes is a metabolic disease that impairs the body's insulin production, leading to unstable blood sugar levels. Chronic hyperglycemia (low blood sugar) accelerates the aging of diabetic patients' organs and, in severe cases, causes organ dysfunction or failure [5]. According to the World Health Organization, diabetes has become the third most serious threat to human health [6]. The Global Diabetes Map (released by the International Diabetes Federation) shows that the number of diabetic patients aged 20–79 in 2019 was about 463 million, marking a three-fold increase since the year 2000. The World Health Organization estimates that the number of diabetic patients aged 20–79 will reach 700 million by 2045 and has shown that the global annual expenditure on diabetes health is approximately 760 billion US dollars.

The high cost of diabetes treatment not only has a significant impact on patients and their families but also represents a heavy burden to society. In view of the serious harm of diabetes to the world, research on this disease has increased in recent years. While seeking new drugs to treat diabetes, researchers are actively exploring nonpharmaceutical treatment options. Exercise has proven to be an effective means of prevention and treatment of chronic diseases. Additionally, encouraging results have also been observed in exercise-based interventions for patients with diabetes. Researchers have used aerobic and resistance exercises in interventions with diabetic patients; their studies have shown that aerobic and resistance exercises effectively improved patients' level of glucose and lipid metabolism [7, 8].

Tai Chi is a physical and mental exercise that integrates martial arts with mental and breathing exercises. Practicing Ta Chi can effectively improve flexibility, balance, and coordination, while it has also proven effective against heart disease [9], coronary heart disease [10], hypertension [11], and cancer [12]. However, researchers hold different views regarding the effects of Tai Chi on diabetic patients' glucose and lipid metabolism. For example, Xia et al. found that Tai Chi reduced moderately the fasting blood glucose and glycosylated hemoglobin levels of patients with type 2 diabetes (T2D) [13]. Furthermore, Zhou et al. found that Tai Chi had a significant effect on reducing fasting blood glucose, glycosylated hemoglobin, and total cholesterol level of patients with T2D [14]. Additionally, Tang Qing et al. found that Tai Chi reduced fasting blood glucose level and glycosylation in patients with T2D and that hemoglobin, triglycerides, and body weight also exhibited improvements; however, the adjustment of total cholesterol was not statistically significant [15]. Conversely, other studies have noted that Tai Chi had no significant effects on glycosylated hemoglobin in patients with T2D [16, 17].

Therefore, results regarding the effectiveness of Tai Chi in the prevention and treatment of glucose and lipid metabolism in diabetes are inconclusive. Additionally, there is a lack of systematic reviews focused on glucose and lipid metabolism in middle-aged and elderly diabetic patients. Furthermore, prior studies have seldom taken into account multiple variables, such as single-session duration, exercise frequency, and overall duration. Therefore, through this research review, we aimed to explore the influence of Tai Chi on the metabolism of glucose and lipids in middle-aged and elderly diabetic patients and to assess whether there is a scientific basis to support the implementation of Tai Chi interventions for middle-aged and elderly diabetic patients.

## 2. Methods

### 2.1. Data Sources

To identify studies on the effects of Tai Chi on diabetes, we conducted a search of Chinese and English electronic databases, such as Web of Science, PubMed, the Cochrane Library, EMBASE, Google Scholar, CNKI, Weipu, and Wanfang. Chinese search keywords were the following: “Tai Chi,” “diabetes,” “type 1 diabetes,” and “type 2 diabetes.” English search keywords were the following: “Tai Chi,” “Tai Ji,” “Tai Chi Exercise,” “Tai Ji Chuan,” “Tai Ji Quan,” “Tai Chi Chuan,” “Tai Chi Quan,” “Taichi,” “Taichiquan,” and “diabetes,” “diabetes mellitus,” “type 2 diabetes mellitus,” and “type 1 diabetes mellitus.” The upper limit for the publication date of articles was August 1, 2020. Taking the Web of Science as an example, the detailed search strategy is presented in Table 1.

### 2.2. Inclusion Criteria and Exclusion Criteria

#### 2.2.1. Eligibility Criteria

Among our inclusion criteria, we included the following: (1) The randomized controlled trial (RCT) also explored the relationship between Tai Chi and diabetes; (2) the study included at least one outcome measurement of diabetes-related indicators; (3) the study population comprised middle-aged and elderly patients with a diabetes diagnosis; and (4) the article was published no later than August 1, 2020.

Among our exclusion criteria, we included the following: (1) duplicate studies; (2) literature reviews, theories, abstracts, and nonrandomized controlled experiments; (3) the study included patients with severe complications; (4) the article did not provide sufficient result data, while the original data could not be obtained after contacting the author; and (5) studies with poor quality assessment.

#### 2.2.2. Trial Inclusion

Study selection was carried out by two researchers independently. By carefully reading studies' title, abstract, and full text, studies that were not related to the main subject of this review were discarded. If there was any disagreement during the literature review process, the three researchers discussed it and sought a consensus regarding the inclusion of the reviewed study. Finally, the researchers determined which studies were qualified for inclusion in this review.

#### 2.2.3. Trial Quality Assessment

The quality of the studies was assessed according to the Physiotherapy Evidence Database (PEDro) scale [18]. The PEDro scale comprises 11 dimensions: eligibility criteria evaluation, randomization, allocation hiding, similar baseline, participant blinding, instructor blinding, evaluator blinding, a retention rate of over 85%, treatment intention analysis, comparison between groups, and point measurement and variability measurement [9]. Satisfying one of the indicators was counted as a 1-point addition to the score. Since the first item of the PEDro scale is not included in the total score, the total score of the scale is 10 points. A total score of ≥7 indicates high quality, 5-6 points indicate medium quality, and anything lower indicates poor quality [19, 20].

#### 2.2.4. Data Extraction

The coding of the included literature was independently completed by two researchers. The basic information extracted included the name of the first author, the year of publication, the characteristics of the test, the intervention methods, the intervention period, the intervention frequency, and the type of outcome indicators. If two researchers encountered differences in the process of extracting basic information, the three researchers discussed and resolved them together.

### 2.3. Statistical Analysis

To conduct this meta-analysis, we used Stata 14.0 software. Analysis modules included the combined effect size, publication bias analysis, heterogeneity analysis, sensitivity analysis, and subgroup analysis. Since the outcome indicators of studies included in this review were continuous variables, the measurement data are shown as the Weighted Mean Difference (WMD). The included studies were tested for heterogeneity before merging their effect sizes. The heterogeneity test between the studies was performed using the *χ*^2^ test. When the included studies had statistical homogeneity (*I*^2^< 50%), a fixed meta-analysis was performed on the effect model. When the statistical heterogeneity between the included studies was large (*I*^2^≥ 50%), a random-effects model was used for meta-analysis. When *I*^2^ was greater than 75%, it was considered highly heterogeneous. The source and control of heterogeneity adopted the method of subgroup analysis, and publication bias adopted the funnel chart.

## 3. Results

### 3.1. Study Selection

**Figure 1:**
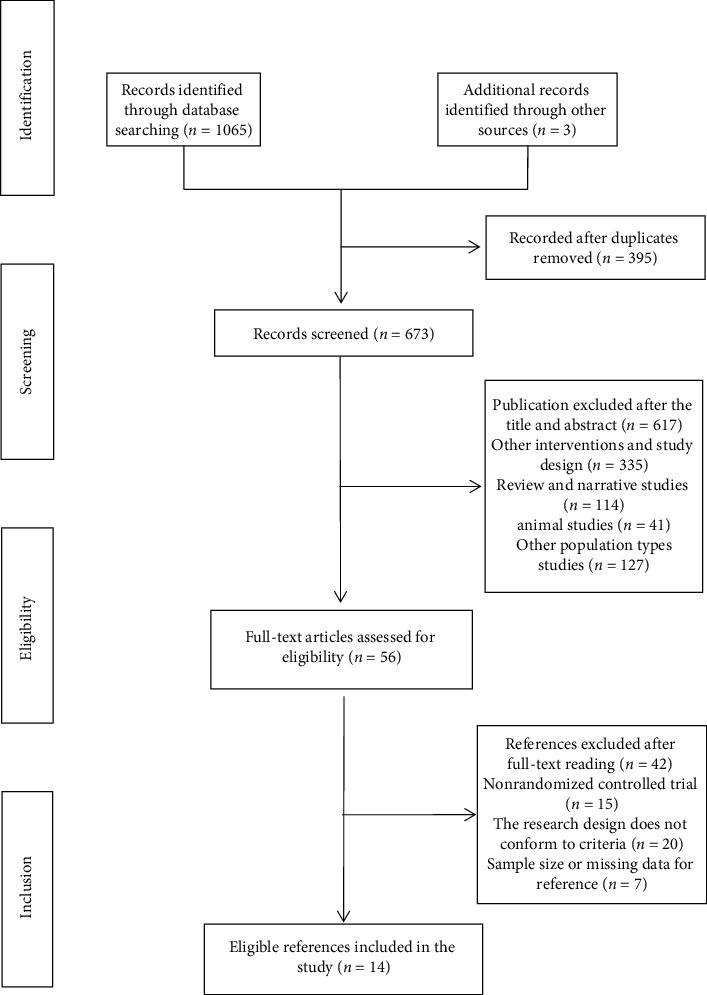
Flow of study selection.

Our database search yielded 1,068 studies. After discarding duplicate documents and reading studies' titles and abstracts to assess their relevance for this review, 56 articles remained. These studies were rescreened after reading the full text. We excluded 15 nonrandomized controlled trial studies, 20 studies with inconsistent research designs, and 7 studies with missing or unavailable data. Thus, 14 articles were finally included (Figure 1), including 10 articles in Chinese and 4 articles in English.

### 3.2. Study Characteristics

All 14 studies (Table 2) were randomized controlled trials. About 64.3% of the articles were published in 2010. The sample sizes ranged from 16 to 108. Twelve studies specified the gender distribution of their samples, which, in total, comprised 285 women and 232 men. The average duration of Tai Chi interventions varied. The shortest duration registered was 8 weeks, and the longest was 24 weeks. The number of exercise sessions per week ranged from 1 to 7, while a single-session duration ranged from 30 to 60 min.

## 4. Methodological Quality

The PEDro scale showed that the methodological quality of the 14 studies ranged from 6 to 8 with an average score of 6.57 (Table 3), indicating that the overall methodological quality of the included studies was good. All 14 studies were randomized controlled trials. We reviewed inclusion conditions, statistical results between groups, point measurement values, and variation measurement values. Two of the studies blinded the assessors of the test, subjects of 8 studies received experimental interventions according to the allocation plan, and 14 studies obtained measurement results.

### 4.1. Influence of Tai Chi on Middle-Aged and Elderly Diabetic Patients

#### 4.1.1. Fasting Blood Glucose

**Figure 2:**
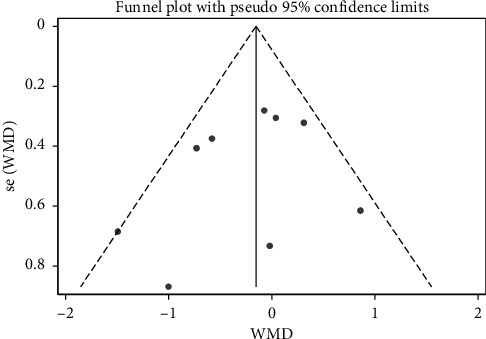
Funnel plot of Tai Chi's effect on fasting blood glucose levels in middle-aged and elderly diabetic patients.

**Figure 3:**
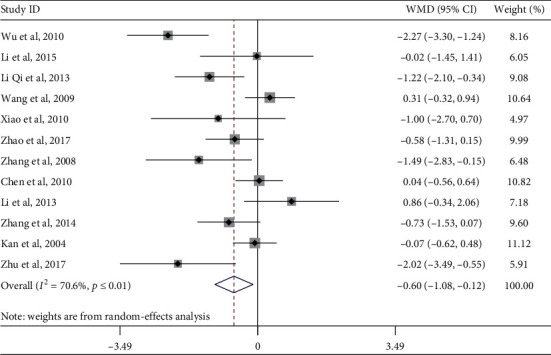
Forest plot of the effects of Tai Chi on fasting blood glucose levels in middle-aged and elderly diabetic patients.

Twelve studies evaluated the effect of Tai Chi on fasting blood glucose in middle-aged and elderly diabetic patients. We used funnel plots to detect publication bias. Three outliers were excluded, namely, the work of Wu et al. (2010), Li Qi et al. (2013), and Zhu et al. (2017). The final funnel diagram is shown in Figure 2. The heterogeneity test of these 12 studies showed heterogeneity (*p* ≤ 0.01, *I*^2^ = 70.6%). Therefore, a random-effects model was used for meta-analysis (Figure 3). The results showed that the improvement effect of Tai Chi on fasting blood glucose levels among elderly diabetic patients was statistically significant (WMD = −0.60, 95% CI [−1.08, −0.12], *p*=0.015).

In order to explore whether the heterogeneity among the included studies was caused by a single study, a sensitivity analysis of heterogeneity was carried out to assess the stability of the overall effect size. After excluding the articles by Wu et al. (2010), Zhu et al. (2017), and Li Qi et al. (2013), a meta-analysis of the remaining nine studies showed that the studies showed heterogeneity in sexual decline (*I*^2^ = 40.3%, *p*=0.099) and combined effect size (WMD = −0.20, 95% CI [−0.57, 0.17], *p*=0.294).

Subgroup analysis was performed on the outcome index of fasting blood glucose levels. We also performed a subgroup analysis of exercise cycle, exercise frequency, and single-session exercise time, according to the research intervention characteristics that could cause heterogeneous differences (Table 4). Among them, the combined effect size of Tai Chi exercises with an exercise period of more than 12 weeks was WMD = −0.78, 95% CI (−1.47, −0.08), *p*=0.029, indicating that practicing Tai Chi over a 12-week period can significantly improve the fasting blood glucose levels of middle-aged and elderly diabetic patients. In the subgroup of single-session exercise time, the combined effect size of Tai Chi sessions lasting longer than 50 minutes was WMD = −1.04, 95% CI (−1.70, −0.37), *p*=0.002, indicating that Tai Chi sessions longer than 50 minutes can significantly improve the fasting blood sugar levels of middle-aged and elderly diabetic patients.

#### 4.1.2. Glycated Hemoglobin

**Figure 4:**
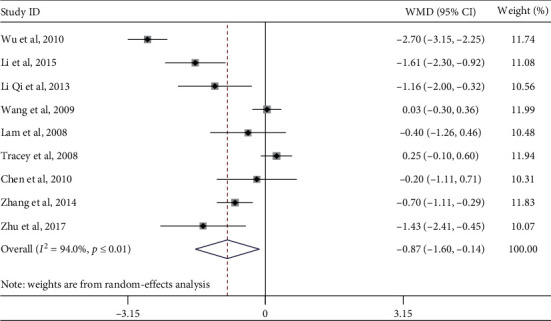
Forest plot of the influence of Tai Chi on glycosylated hemoglobin levels in middle-aged and elderly diabetic patients.

Nine studies evaluated the effect of Tai Chi on glycated hemoglobin levels in middle-aged and elderly diabetic patients. Heterogeneity analysis showed that there was heterogeneity among the included studies (*p* ≤ 0.01, *I*^2^ = 94.0%); therefore, a randomized meta-analysis of the effect model (Figure 4) showed that Tai Chi improved the glycosylated hemoglobin of middle-aged and elderly diabetic patients, and the difference was statistically significant (WMD = −0.87, 95% CI [−1.60, −0.14], *p*=0.019).

In order to explore whether the heterogeneity among the included studies was caused by a single study, a sensitivity analysis of heterogeneity was carried out to assess the stability of the overall effect size. We found that the articles by Wu et al. (2010), Li et al. (2015), Tracey et al. (2008), and Wang et al. (2009) were quite heterogeneous. After excluding these four articles, five studies remained. The meta-analysis showed that the heterogeneity decreased after excluding the aforementioned four studies: *I*^2^ = 17.5%, *p*=0.303, WMD = −0.75, 95% CI (−1.10, −0.39), *p* ≤ 0.01.

A subgroup analysis was performed on the outcome indices of glycosylated hemoglobin, exercise period, exercise frequency, and single-session exercise time, according to the research intervention characteristics that could cause heterogeneous differences (Table 4). In the subgroup of exercise frequency, the combined effect size of practicing Tai Chi more than 3 times per week was WMD = −0.91, 95% CI (−1.56, −0.25), *p*=0.007, indicating that practicing Tai Chi more than three times per week can significantly improve the glycosylated hemoglobin levels of middle-aged and elderly diabetic patients.

#### 4.1.3. Total Cholesterol

**Figure 5:**
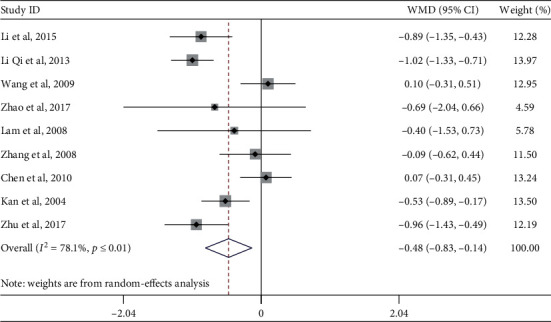
Forest plot of the effect of Tai Chi on total cholesterol levels among middle-aged and elderly diabetic patients.

Nine studies evaluated the effect of Tai Chi on total cholesterol levels among middle-aged and elderly diabetic patients. Heterogeneity analysis showed that there was heterogeneity among the included studies (*p* ≤ 0.01, *I*^2^ = 78.1%). Subsequently, a randomized meta-analysis of the effect model (Figure 5) showed that Tai Chi improved the total cholesterol levels of middle-aged and elderly diabetic patients and that the difference was statistically significant (WMD = −0.48, 95% CI [−0.83, −0.14], *p*=0.006).

In order to explore whether the heterogeneity among the included studies was caused by a single study, a sensitivity analysis of heterogeneity was carried out to assess the stability of the overall effect size and found significant heterogeneity in the articles by Li Qi et al. (2013), Wang et al. (2009), and Chen et al. (2010). After excluding these three articles, a meta-analysis of the remaining six studies showed decreased heterogeneity (*I*^2^ = 34.2%, *p*=0.180), WMD = −0.62, 95% CI (−0.90, −0.33), *p* ≤ 0.01.

A subgroup analysis was performed on the outcome indices of total cholesterol, exercise period, exercise frequency, and single-session exercise time. Each subgroup was analyzed according to the research intervention characteristics that could cause heterogeneous differences (Table 4). In the exercise cycle subgroup, the combined effect size of Tai Chi practice lasting no longer than 12 weeks was WMD = −0.61, 95% CI (−1.11, −0.11), and *p*=0.016. This indicates that Tai Chi practice lasting no longer than 12 weeks significantly improved the total cholesterol level of middle-aged and elderly diabetic patients, which is inconsistent with the results of previous studies. This could be because of human exercise stress and adaptability. Practicing Tai Chi for no longer than 12 weeks promoted the uptake of fatty acids by muscle tissue and accelerated the transport and degradation of total cholesterol. Additionally, practicing Tai Chi for more than 12 weeks may prompt the human body to adapt to the physical demands of exercise, leading to a plateau in stress level and a decrease in the efficiency of adipose tissue mobilization. In the exercise frequency subgroup, the combined effect size of practicing Tai Chi more than three times per week was WMD = −0.58, 95% CI (−0.94, −0.22), *p*=0.002, indicating that practicing Tai Chi more than three times per week significantly improves the total cholesterol levels of middle-aged and elderly diabetic patients. Regarding single-session exercise time, the combined effect amount of Tai Chi sessions lasting longer than 50 minutes was WMD = −0.40, 95% CI (−0.77, −0.03), *p*=0.036, indicating that Tai Chi sessions lasting longer than 50 minutes can significantly improve the total cholesterol of middle-aged and elderly diabetic patients.

#### 4.1.4. Triglycerides

**Figure 6:**
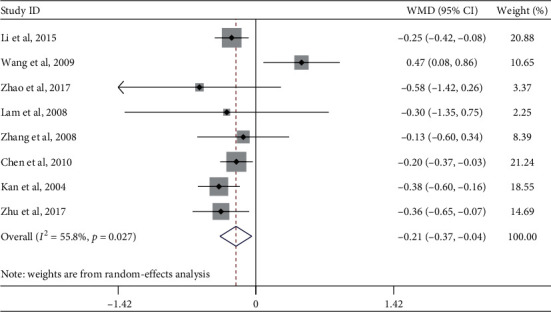
Forest plot of Tai Chi influence on triglyceride in middle-aged and elderly diabetic patients.

Eight studies evaluated the effect of Tai Chi on triglyceride levels in middle-aged and elderly patients with diabetes. Heterogeneity analysis showed that there was heterogeneity among the included studies (*p*=0.027, *I*^2^ = 55.8%). A meta-analysis of the random-effects model (Figure 6) showed that Tai Chi improved triglyceride levels of middle-aged and elderly diabetic patients and that the difference was statistically significant (WMD = −0.21, 95% CI [−0.37, −0.04], *p*=0.014).

In order to explore whether the heterogeneity among the included studies was caused by a single study, a sensitivity analysis of heterogeneity was carried out to assess the stability of its overall effect size. Wang et al.'s (2009) study was relatively heterogeneous. After excluding this article, a meta-analysis of the remaining seven studies showed that the heterogeneity decreased (*I*^2^ = 0.0%, *p*=0.814), WMD = −0.27, 95% CI (−0.37, −0.18), *p* ≤ 0.01.

A subgroup analysis was performed on the outcome indices of total cholesterol, exercise period, exercise frequency, and single-session exercise time according to the research intervention characteristics that could cause heterogeneous differences (Table 4). The results showed that the three different subgroups found no significant improvement in triglyceride levels in middle-aged and elderly diabetic patients.

#### 4.1.5. High-Density Lipoprotein Cholesterol

**Figure 7:**
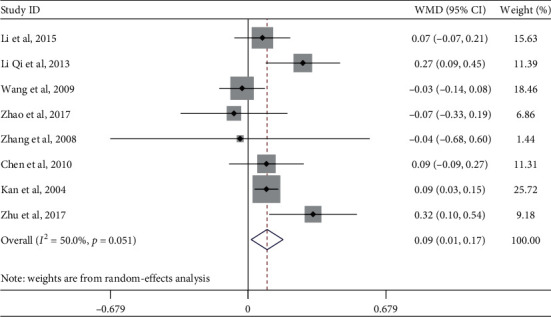
Forest plot of Tai Chi influence on high-density lipid-cholesterol in middle-aged and elderly diabetic patients.

Eight studies evaluated the effect of Tai Chi on high-density lipoprotein (HDL) cholesterol in middle-aged and elderly diabetic patients. Heterogeneity analysis showed that these studies exhibited heterogeneity (*p*=0.051, *I*^2^ = 50.0%). Therefore, a random-effects model was used for meta-analysis (Figure 7), which showed that Tai Chi did not significantly improve HDL cholesterol levels in middle-aged and elderly diabetic patients (WMD = 0.09, 95% CI [−0.01, 0.17], *p*=0.136).

To explore whether the heterogeneity among the included studies was caused by a single study, a sensitivity analysis of heterogeneity was carried out to assess the stability of the overall effect size. Zhu et al.'s (2017) article exhibited relatively large heterogeneity. After excluding this article, a meta-analysis of the remaining seven articles showed that the heterogeneity decreased (*I*^2^ = 35.1%, *p*=0.160), WMD = 0.07, 95% CI (0.00, 0.14), *p*=0.045.

Although practicing Tai Chi did not significantly improve HDL cholesterol in middle-aged and elderly diabetic patients, this review further explored the influence of different factors on HDL cholesterol, hoping to provide a scientific basis for future clinical exercise interventions for diabetes. According to the research intervention characteristics that could cause heterogeneous differences, we analyzed exercise period, exercise frequency, and single-session exercise time by subgroup analysis (Table 4). Tai Chi's improvement of protein cholesterol levels was not evident.

#### 4.1.6. Low-Density Lipoprotein Cholesterol

**Figure 8:**
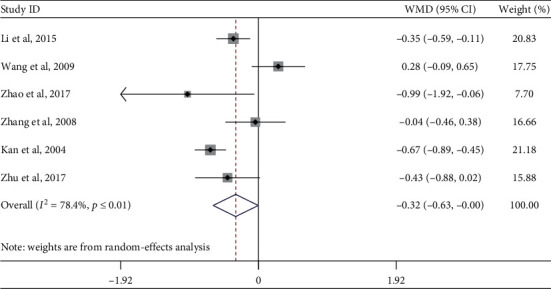
Forest plot of Tai Chi influence on low-density lipid-cholesterol in middle-aged and elderly diabetic patients.

Six studies evaluated the effect of Tai Chi on low-density lipoprotein cholesterol levels in middle-aged and elderly people. Heterogeneity analysis showed that there was heterogeneity among the included studies (*p* ≤ 0.01, *I*^2^ = 78.4%). Therefore, a random-effects model was used for meta-analysis (Figure 8), showing that Tai Chi improved the low-density lipoprotein cholesterol levels of middle-aged and elderly diabetic patients, and the difference was statistically significant (WMD = −0.32, 95% CI [−0.63,− 0.00], *p*=0.050).

In order to explore whether the heterogeneity among the included studies was caused by a single study, a sensitivity analysis of heterogeneity was carried out to assess the stability of the overall effect size. We found that the articles by Zhao et al. (2017) and Wang et al. (2009) were relatively heterogeneous. After excluding these two articles, the meta-analysis of the remaining four studies showed that the heterogeneity decreased (*I*^2^ = 24.6%, *p*=0.264), WMD = −0.33, 95% CI (−0.57, −0.10), *p*=0.006.

A subgroup analysis was performed for the outcome indicator of low-density lipoprotein cholesterol. According to the research intervention characteristics that could cause heterogeneous differences, subgroup analysis was performed on the exercise cycle and single-session exercise time variables (Table 4). In the subgroup, the combined effect size of Tai Chi sessions lasting longer than 50 min was WMD = −0.47, 95% CI [−0.83, −0.12], *p*=0.008, indicating that practicing Tai Chi in +50-minute sessions can significantly improve low-density lipoprotein cholesterol in middle-aged and elderly diabetic patients.

## 5. Discussion

The International Diabetes Federation has stated that a healthy diet and regular physical exercise play a large role in preventing type 2 diabetes and controlling blood sugar [35]. Studies have found that engaging in aerobic and resistance exercise for 30 minutes every day can reduce the risk of diabetes by 43% [36]. Furthermore, Peng et al. found that the longer the diabetic patients persisted in performing exercise (specifically, Baduanjin qigong), the greater the decrease in glycosylated hemoglobin they exhibited [7]. Moreover, the research by Zhang et al. pointed out that a 14-week Tai Chi exercise can significantly improve blood glucose, glycosylated serum protein, triglyceride, and other related indicators in elderly women with type 2 diabetes [27].

Some scholars have conducted a review on Taijiquan's improvement of glucose and lipid metabolism in diabetic patients, but some reviews [14, 37, 38] only explored the effect of Taijiquan on the blood sugar of diabetic patients without involving the effect of Taijiquan on diabetic patients' blood lipids. Studies have confirmed that, in addition to abnormal blood glucose, the factors which cause cardiovascular complications in diabetic patients are important, so attention should also be paid to their blood lipids. The second part of the review [16, 38] included studies that used nonrandomized controlled trials, which would increase the degree of bias in the research and affecting its results [13–17, 37, 39, 40]. The intervention methods included in the study were Qigong, Dayuan Hypoglycemic (Tai Chi) Exercise, and Tai Chi Soft Ball. Like Dayuan Hypoglycemic (Tai Chi) Exercise, Tai Chi Soft ball is created through Tai Chi thinking, but it is still different from Tai Chi exercise. It is difficult to sink the dantian and guide the qi with the mind, and it is even more difficult to coordinate the heart, mind, and qi. This is bound to have a certain impact on the final intervention effect, which then affects the system review's objectivity. Only one study [37] explored the dose effect of Tai Chi on the blood sugar of patients with type 2 diabetes according to different types of exercise and total exercise volume (single exercise time  ^*∗*^ exercise days per week  ^*∗*^ exercise weeks); instead, the total exercise volume was used. The dose effect on glucose and lipid metabolism in diabetic patients is insufficient as the same total exercise amount has different effects. This study did not explore the effect of Tai Chi exercises when different times and frequencies were used or the effect of a single exercise period on diabetic patients' blood sugar. The influence of Tai Chi on diabetic patients' blood lipids has not been explored.

The innovations of this article are as follows: (1) Conduct a subgroup analysis considering the dimensions of “exercise cycle,” “exercise frequency,” and “single exercise time” and deeply explore the dose effect of Tai Chi on the glucose and lipid metabolism of diabetic patients. (2) All research methods in the study are randomized controlled trials, and the experimental group uses Tai Chi as an intervention technique. (3) The research on diabetic patients in this article includes not only blood sugar (fasting blood glucose and glycosylated hemoglobin) but also blood lipids (total cholesterol, triglycerides, low-density lipoprotein cholesterol, and high-density lipoprotein cholesterol) and systematically studied Tai Chi effects on glucose and lipid metabolism in diabetic patients.

This research review shows that Tai Chi can significantly improve fasting blood glucose and glycosylated hemoglobin in middle-aged and elderly diabetic patients, which is consistent with the results of previous studies [13–15, 37, 39]. Additionally, we found that Tai Chi significantly improved triglycerides, total cholesterol, and low-density lipoprotein levels in middle-aged and elderly diabetic patients, but it had no significant effect on high-density lipoprotein cholesterol. Similarly, Xia et al. found that Tai Chi significantly improved triglyceride levels in diabetic patients [13]. Additionally, the research by Liu Yongjin et al. found that Tai Chi significantly improved triglycerides, total cholesterol, and low-density lipoprotein levels in diabetic patients [39].

Among the outcome indicators of glucose metabolism, Tai Chi improved fasting blood glucose and glycosylated hemoglobin levels in middle-aged and elderly diabetic patients. The reasons for this may be that (1) Tai Chi can promote skeletal muscle's uptake and use of glucose in the blood and increase the activity of insulin in the body to convert blood sugar. The activity of the glucose transporter on the skeletal muscle cell membrane is to further improve the blood glucose concentration in the body [22]; (2) Tai Chi has both the advantages and characteristics of oxygen and resistance exercise. Middle-aged and elderly diabetic patients maintain a standing posture for a long time during the practice of Tai Chi. Therefore, leg muscle strength is increased and the cross section of muscle fibers is enlarged, which causes the leg muscles to grow. (3) Absorption and utilization of blood sugar: Practicing Tai Chi engages the whole body in the exercise, especially small muscle groups and peripheral tissues, and promotes the absorption of glucose [41].

Additionally, Tai Chi can improve total cholesterol and triglyceride levels in middle-aged and elderly diabetic patients. The reason for this may be that exercise increases the activity of lipoprotein lipase in the body, which promotes the hydrolysis of triglycerides in the body. Lipoprotein lipase promotes lipolysis in the body after an increase in activity, reducing total cholesterol levels and triglycerides in plasma, accelerating the conversion and degradation of triglycerides, and increasing the body's ability to remove cholesterol in the blood [42]. Second, exercise increases the permeability of skeletal muscle capillaries, increases the surface area of the capillary endothelium, increases lipoprotein lipase activity in skin cells, and promotes the decomposition of total cholesterol and triglycerides in plasma. A decrease in low-density lipoprotein cholesterol is related to activity in low-density lipoprotein (LDL) receptors and ApoB levels. When LDL receptor activity increases or ApoB levels decrease, serum low-density lipoprotein cholesterol levels decrease [43].

The possible reasons why the effect of Tai Chi on HDL cholesterol in middle-aged and elderly diabetic patients was not statistically significant (compared with the control group) are exercise intensity and gender. Studies have found that moderate-intensity (50% VO_2_max) and greater-intensity (70% VO_2_max) exercise are more suitable for elderly diabetic patients [44]. Five of the studies we reviewed did not specify exercise intensity, while two studies specified having implemented low-intensity exercise. An unsuitable exercise intensity cannot guarantee that fat is the main component. As the functional substance of HDL, cholesterol did not change significantly. Furthermore, Xiao et al. implemented an aerobic exercise intervention for 12 weeks and found that the level of high-density lipoprotein cholesterol in men's plasma increased significantly, while the level of HDL cholesterol in women's plasma had not changed significantly [45].

This review has some limitations. First, the subjects included in the study have a large difference in their illness duration. Existing studies have shown that the risk of complications from diabetes, including strokes, will increase as the duration of disease increases. Increasing duration also increases the difficulty of controlling diabetes, which may be one of the causes of heterogeneity. Second, the control group included in the study received different intervention methods, mainly divided into three types: drug therapy, exercise intervention, and drug therapy combined with exercise intervention. Different intervention methods have different effects in terms of controlling the subjects' condition, which will affect the research results. Third, the postures and familiarity of the participants in the included study of Taijiquan are not explained. Taijiquan training in different postures will affect the cardiovascular function and athletic ability of the participants. The inconsistency in proficiency of Taijiquan will interfere with the subjects as their mind and breath of the participants during the practice have a certain impact on the effect of the intervention. Fourth, among the elements of Tai Chi intervention, differences in exercise intensity, exercise time, exercise frequency, and exercise items also have different effects on the glucose and lipid metabolism of the subjects, thereby affecting the authenticity of the research results.

## 6. Conclusions

Tai Chi can significantly improve fasting blood glucose, glycosylated hemoglobin, total cholesterol, triglycerides, and low-density lipoprotein cholesterol metabolism in middle-aged and elderly diabetic patients, but it has no significant effect on high-density lipoprotein cholesterol metabolism; practicing Tai Chi in sessions lasting longer than 50 minutes (at least three times per week, for at least 12 weeks) can effectively improve glucose and lipid metabolism in middle-aged and elderly diabetic patients. However, several other factors affect glucose and lipid metabolism; therefore, further high-quality research is needed.

## Figures and Tables

**Table 1:** Web of Science search strategy.

Order	Search terms
#1	Tai Chi
#2	Tai Ji
#3	Tai Chi exercise
#4	Tai Ji Chuan
#5	Tai Ji Quan
#6	Tai Chi Chuan
#7	Tai Chi Quan
#8	Taichi
#9	Taichiquan
#10	#1 OR #2 OR #3 OR #4 OR #5 OR #6 OR #7 OR #8 OR #9
#11	Diabetes
#12	Diabetes mellitus
#13	Type 2 diabetes mellitus
#14	Type 1 diabetes mellitus
#15	#11 OR #12 OR #13 OR #14
#16	#10 AND #15

**Table 2:** Characteristics of the studies included in the meta-analysis.

References	F/M	Sample size (attrition rate)	Population	Course of disease	Duration (week)	Time (min)	Frequency (weekly)	Motion intensity	Intervention program	Outcome measured
Wu et al. [21], China, 2010	25/15	40 (0%)	M	EG: 1.35 (0.62) CG: 1.36 (0.7)	24	60	3	Moderate	EG : TC (Yang style) + DT CG : DT	FBG and HbA1c
Li et al. [22], China, 2015	NR	100 (0%)	E	EG: 7.83 (2.16) CG: 8.14 (3.19)	24	40–50	7	NR	EG : TC (Chen style) CG : AE	FBG, HbA1c, TC, TG, HDL-C, and LDL-C
Li Qi et al. [23], China, 2013	NR	108 (19.4%)	M + E	EG: 6.95 (3.63) CG: 7.22 (4.14)	12	30	7	NR	EG : TC (Yang style) + DT CG : DT	FBG, HbA1c, TC, and HDL-C
Wang et al. [24], China, 2009	24/30	54 (0%)	M	EG: 1.35 (0.62) CG: 1.36 (0.7)	24	30–50	5	Moderate	EG : TC (Yang style) CG : AE	FBG, HbA1c, TC, TG, HDL-C, and LDL-C
Xiao et al. [25], China, 2010	10/14	24 (0%)	M	NR	24	60	6	Moderate	EG : TC (Yang style) + DT CG : DT	FBG
Zhao et al. [26], China, 2017	0/16	16 (11%)	M	NR	16	60	7	Moderate	EG : TC (Chen style) + DT CG : DT	FBG, TC, TG, HDL-C, and LDL-C
Zhang et al. [27], China, 2008	20/0	20 (5%)	M + E	NR	14	60	5	Low	EG : TC (Yang style) + DT CG : CE + DT	FBG, TC, TG, HDL-C, and LDL-C
Lam et al. [28], Australia, 2008	29/24	53 (17%)	M + E	NR	24	60	1	NR	EG : TC (other types) CG : CE	HbA1c, TC, and TG
Tracey et al. [29], Australia, 2008	30/8	38 (2.6%)	E	NR	16	60	2	Low	EG : TC (other types) CG : MM	HbA1c
Chen et al. [30], China, 2010	59/45	104 (9%)	M + E	EG: 7.8 (3.1) CG: 8.5 (3.5)	12	60	3	NR	EG : TC (Chen style) + DT CG : AE + DT	FBG, HbA1c, TC, TG, HDL-C, and LDL-C
Li et al. [31], China, 2013	24/36	60 (0%)	M + E	NR	8	45	7	Moderate	EG : TC (other types) + UC CG : UC	FBG
Zhang et al. [32], China, 2014	31/9	40 (0%)	M + E	NR	14	60	3	Moderate	EG : TC (Yang style) CG : AE	FBG and HbA1c
Kan et al. [33], China, 2004	23/25	48 (0%)	M	NR	12	60	7	NR	EG : TC (Yang style) CG : AE	FBG, TC, TG, HDL-C, and LDL-C
Zhu et al. [34], China, 2017	10/10	20 (0%)	M + E	EG: 5.15 (2.53) CG: 5.30 (2.65)	12	60	5	Moderate	EG : TC (Chen style) CG : UC	FBG, HbA1c, TC, TG, HDL-C, and LDL-C

*Note.* EG = exercise group; CG = control group; NR = not reported; outcome measured (FBG = fasting blood glucose; HbA1c = glycosylated hemoglobin; TC = total cholesterol; TG = triglyceride; HDL-C = high-density lipoprotein cholesterol; and LDL-C = low-density lipoprotein cholesterol); intervention program (TC = Tai Chi; DT = drug therapy; UC = usual care; AE = aerobic exercise; MM = mixed movement; and CE = conventional exercise); and population type (M = middle-aged; *E* = elderly).

**Table 3:** Study-quality assessment of eligible randomized controlled trials.

Reference	Item 1	Item 2	Item 3	Item 4	Item 5	Item 6	Item 7	Item 8	Item 9	Item 10	Item 11	Score
Wu et al. (2010) [21]	1	1	0	1	0	0	0	1	1	1	1	7
Li et al. (2015) [22]	1	1	0	1	0	0	0	1	1	1	1	7
Li Qi et al. (2013) [23]	1	1	0	1	0	0	0	1	0	1	1	6
Wang et al. (2009) [24]	1	1	0	1	0	0	0	1	1	1	1	7
Xiao et al. (2010) [25]	1	1	0	1	0	0	0	0	1	1	1	6
Zhao et al. (2017) [26]	1	1	0	1	0	0	0	1	0	1	1	6
Zhang et al. (2008) [27]	1	1	0	1	0	0	0	1	0	1	1	6
Lam et al. (2008) [28]	1	1	0	1	0	0	1	1	0	1	1	7
Tracey et al. (2008) [29]	1	1	0	1	1	0	1	1	0	1	1	8
Chen et al. (2010) [30]	1	1	0	1	0	0	0	1	0	1	1	6
Li et al. (2013) [31]	1	1	0	1	0	0	0	1	1	1	1	7
Zhang et al. (2014) [32]	1	1	0	1	0	0	0	1	1	1	1	7
Kan et al. (2004) [33]	1	1	0	0	0	0	0	1	1	1	1	6
Zhu et al. (2017) [34]	1	1	0	0	0	0	0	1	1	1	1	6

*Note.* Item 1 = eligibility criteria; item 2 = random sequence; item 3 = allocation concealment; item 4 = similar at baseline; item 5 = subjects blinded; item 6 = therapists blinded; item 7 = assessors blinded; item 8 = <15% dropouts; item 9 = intention-to-treat analysis; item 10 = between-group comparison; item 11 = point measures and variability data; 1 = meets the criteria; 0 = did not meet the criteria.

**Table 4:** Subgroup analysis of Tai Chi on glucose and lipid metabolism in diabetic patients.

Outcomes	Group	Subgroup	*N*	WMD	95%CI	*p*	*I* ^2^ (%)	(WMD) *p* value
FBG	Duration	≤12 weeks	5	−0.39	−1.12, 0.34	0.005	73.00	0.293
		>12 weeks	7	−0.78	−1.47, −0.08	0.002	71.10	0.029
	Frequency	≤3 times/week	4	−0.67	−1.51, 0.17	0.001	82.10	0.12
		>3 times/week	8	−0.57	−1.22, 0.08	0.004	66.10	0.084
	Time	≤50 min	5	−0.06	−0.67, 0.55	0.036	61.00	0.84
		>50 min	7	−1.04	−1.70, −0.37	0.004	69.00	0.002
HbA1c	Duration	≤12 weeks	4	−0.82	−1.24, −0.39	0.243	28.20	≤0.01
		>12 weeks	5	−0.88	−2.07, 0.30	≤0.01	96.90	0.144
	Frequency	≤3 times/week	4	−0.77	−2.44, 0.89	≤0.01	97.10	0.363
		>3 times/week	5	−0.91	−1.56, −0.25	≤0.01	84.90	0.007
	Time	≤50 min	3	−0.88	−2.02, 0.27	≤0.01	90.70	0.132
		>50 min	6	−0.87	−1.91, 0.18	≤0.01	95.30	0.103
TC	Duration	≤12 weeks	4	−0.61	−1.11, −0.11	≤0.01	85.80	0.016
		>12 weeks	5	−0.34	−0.81, 0.13	0.027	63.50	0.159
	Frequency	≤3 times/week	2	0.02	−0.34, 0.38	0.441	0.00	0.902
		>3 times/week	7	−0.58	−0.94, −0.22	≤0.01	76.80	0.002
	Time	≤50 min	3	−0.61	−1.31, 0.10	≤0.01	89.80	0.091
		>50 min	6	−0.4	−0.77, −0.03	0.021	62.40	0.036
TG	Duration	≤12 weeks	3	−0.28	−0.40, −0.16	0.369	0.00	≤0.01
		>12 weeks	5	−0.09	−0.45, 0.27	0.018	66.50	0.625
	Frequency	≤3 times/week	2	−0.2	−0.38, −0.04	0.854	0.00	0.016
		>3 times/week	6	−0.19	−0.42, 0.04	0.008	68.10	0.098
	Time	≤50 min	2	0.09	−0.62, 0.79	0.001	90.70	0.808
		>50 min	6	−0.28	−0.40, −0.17	0.719	0.00	≤0.01
HDL-C	Duration	≤12 weeks	4	0.17	0.05, 0.28	0.072	57.20	0.004
		>12 weeks	4	0.00	−0.08, 0.08	0.67	0.00	0.967
	Time	≤50 min	3	0.09	−0.07, 0.25	0.024	73.30	0.268
		>50 min	3	0.11	0.01, 0.20	0.208	32.00	0.038
LDL-C	Duration	≤12 weeks	2	−0.62	−0.82, −0.43	0.349	0.00	≤0.01
		>12 weeks	4	−0.17	−0.57, 0.23	0.011	73.20	0.407
	Time	≤50 min	2	−0.05	−0.67, 0.56	0.005	87.30	0.868
		>50 min	4	−0.47	−0.83, −0.12	0.047	62.30	0.008

## Data Availability

The (continuous) data supporting this meta-analysis are from previously reported studies and datasets, which have been cited. The processed data are available in the supplementary files.

## Supplementary Materials

Supplementary Materials The data of this study are derived from 14 published documents.
