# Supplementary material for: A Meta-Analysis of the Effects of Tai Chi on Glucose and Lipid Metabolism in Middle-Aged and Elderly Diabetic Patients: Evidence from Randomized Controlled Trials
Source: Evid Based Complement Alternat Med. 2021 Mar 22;2021:6699935. doi: 10.1155/2021/6699935 (PMC8007338; doi:10.1155/2021/6699935)
Supplement: Supplementary Materials — The data of this study are derived from 14 published documents. [file 6699935.f1.docx]

**Supplementary File**

The data of this study are derived from 14 published documents. The data summary table is as follows

| **Reference** | **Year** | **experimental group number** | **Control group number** | **FBG** | | | | **HbAc1** | | | | **TC** | | | | **TG** | | | | **HDL-C** | | | | **LDL-C** | | | |
| --- | --- | --- | --- | --- | --- | --- | --- | --- | --- | --- | --- | --- | --- | --- | --- | --- | --- | --- | --- | --- | --- | --- | --- | --- | --- | --- | --- |
|  |  |  |  | **Experimental group** | | **Control group** | | **experimental group** | | **Control group** | | **experimental group** | | **Control group** | | **experimental group** | | **Control group** | | **experimental group** | | **Control group** | | **experimental group** | | **Control group** | |
|  |  |  |  | **Mean** | **SD** | **Mean** | **SD** | **Mean** | **SD** | **Mean** | **SD** | **Mean** | **SD** | **Mean** | **SD** | **Mean** | **SD** | **Mean** | **SD** | **Mean** | **SD** | **Mean** | **SD** | **Mean** | **SD** | **Mean** | **SD** |
| Wu et al.  China | 2010 | 20 | 20 | 7.64 | 1.91 | 9.91 | 1.37 | 7.63 | 0.55 | 10.33 | 0.87 |  |  |  |  |  |  |  |  |  |  |  |  |  |  |  |  |
| Li et al China. | 2015 | 50 | 50 | 7.02 | 3.04 | 7.04 | 4.19 | 3.98 | 1.36 | 5.59 | 2.08 | 4.82 | 1.03 | 5.71 | 1.31 | 1.92 | 0.52 | 2.17 | 0.35 | 1.59 | 0.34 | 1.52 | 0.36 | 2.91 | 0.64 | 3.26 | 0.56 |
| Li Qi et al China | 2013 | 43 | 44 | 6.53 | 1.64 | 7.75 | 2.47 | 7.37 | 1.58 | 8.53 | 2.36 | 4.06 | 0.7 | 5.08 | 0.79 |  |  |  |  | 1.35 | 0.46 | 1.08 | 0.41 |  |  |  |  |
| Wang et al.  China | 2009 | 28 | 26 | 7.23 | 1.21 | 6.92 | 1.15 | 6.92 | 0.56 | 6.89 | 0.67 | 5.07 | 0.87 | 4.97 | 0.64 | 2.05 | 0.71 | 1.58 | 0.76 | 1.2 | 0.19 | 1.23 | 0.23 | 3.18 | 0.82 | 2.9 | 0.56 |
| Xiao et al. China | 2010 | 12 | 12 | 7.6 | 2.8 | 8.6 | 1.1 |  |  |  |  |  |  |  |  |  |  |  |  |  |  |  |  |  |  |  |  |
| Zhao et al.  China | 2017 | 8 | 8 | 6.36 | 0.67 | 6.94 | 0.82 |  |  |  |  | 4.79 | 1.36 | 5.48 | 1.39 | 1.49 | 0.86 | 2.07 | 0.85 | 1.09 | 0.22 | 1.16 | 0.31 | 2.63 | 0.55 | 3.62 | 1.23 |
| Lam et al. Australia | 2008 | 24 | 20 |  |  |  |  | 8.1 | 1.4 | 8.5 | 1.5 | 3.1 | 1.8 | 3.5 | 2 | 1.4 | 1.6 | 1.7 | 1.9 |  |  |  |  |  |  |  |  |
| Zhang et al.  China | 2008 | 10 | 9 | 8.13 | 2.15 | 9.62 | 0.242 |  |  |  |  | 4.42 | 0.22 | 4.51 | 0.78 | 1.06 | 0.56 | 1.19 | 0.49 | 1.44 | 0.47 | 1.48 | 0.87 | 2.48 | 0.38 | 2.52 | 0.53 |
| Tracey et al. Australia | 2008 | 17 | 20 |  |  |  |  | 7.03 | 0.5 | 6.78 | 0.6 |  |  |  |  |  |  |  |  |  |  |  |  |  |  |  |  |
| Chen et al.  China | 2010 | 50 | 44 | 9.96 | 1.23 | 9.92 | 1.67 | 8.3 | 2.2 | 8.5 | 2.3 | 4.89 | 0.82 | 4.82 | 1.03 | 1.93 | 0.38 | 2.13 | 0.44 | 1.16 | 0.46 | 1.07 | 0.45 |  |  |  |  |
| Li et al.  China, | 2013 | 30 | 30 | 6.07 | 2.33 | 5.21 | 2.43 |  |  |  |  |  |  |  |  |  |  |  |  |  |  |  |  |  |  |  |  |
| Zhang et al. China | 2014 | 20 | 20 | 7.18 | 1.33 | 7.91 | 1.24 | 7.08 | 0.63 | 7.78 | 0.69 |  |  |  |  |  |  |  |  |  |  |  |  |  |  |  |  |
| Kan et al.  China | 2004 | 26 | 22 | 8.14 | 0.88 | 8.21 | 1.04 |  |  |  |  | 6.01 | 0.82 | 6.54 | 0.4 | 1.98 | 0.34 | 2.36 | 0.41 | 1.11 | 0.09 | 1.02 | 0.11 | 3.31 | 0.35 | 3.98 | 0.41 |
| Zhu et al. China | 2017 | 10 | 10 | 7.48 | 2.13 | 9.5 | 1.03 | 6.18 | 1.01 | 7.61 | 1.21 | 4.39 | 0.65 | 5.35 | 0.39 | 1.43 | 0.42 | 1.79 | 0.21 | 1.51 | 0.22 | 1.19 | 0.27 | 2.97 | 0.55 | 3.4 | 0.48 |

Since the data unit of Zhao et al. and Chen et al is mg/dL, and the data unit of the other 12 studies is mmol/L, in order to better merge the data, this paper unified the unit to mmol/L, so Zhao et al The data of .China and Chen et al.China have been adjusted, and the unit adjustments are as follows: glucose (mmol/L=18mg/dL), total cholesterol (0.0258mmol/L=mg/dL), triglycerides (0.0113mmol/ L=mg/dL), high-density lipoprotein cholesterol (0.0258mmo/L=mg/dl), low-density lipoprotein cholesterol (0.0259mmol/L=mg/dl)
